# Transformed mucosa‐associated lymphoid tissue lymphomas: A single institution retrospective study including polymerase chain reaction‐based clonality analysis

**DOI:** 10.1111/bjh.15953

**Published:** 2019-05-24

**Authors:** Barbara Kiesewetter, Wolfgang Lamm, Werner Dolak, Julius Lukas, Marius E. Mayerhoefer, Michael Weber, Ana‐Iris Schiefer, Christoph Kornauth, Günther Bayer, Ingrid Simonitsch‐Klupp, Markus Raderer

**Affiliations:** ^1^ Department of Medicine I Clinical Division of Oncology Medical University of Vienna Vienna Austria; ^2^ Department of Medicine III Clinical Division of Gastroenterology and Hepatology Medical University of Vienna Vienna Austria; ^3^ Department of Ophthalmology and Optometry Medical University of Vienna Vienna Austria; ^4^ Division of Nuclear Medicine Department of Biomedical Imaging and Image‐guided Therapy Medical University of Vienna Vienna Austria; ^5^ Department of Biomedical Imaging and Image‐guided Therapy Medical University of Vienna Vienna Austria; ^6^ Department of Pathology Medical University of Vienna Vienna Austria

**Keywords:** MALT lymphoma, indolent lymphoma, histological transformation, clonality analysis, diffuse large B‐cell lymphoma

## Abstract

Given the lack of consistent data regarding the clinico‐pathological features and clonal lymphomagenesis of patients with mucosa‐associated lymphoid tissue (MALT) lymphoma and histological transformation (HT), we have systematically analysed 379 patients (32% gastric, 68% extra‐gastric; median follow‐up 52 months) diagnosed with HT at the Medical University Vienna 1999–2017, and reassessed tissues of identified patients by polymerase chain reaction (PCR)‐based clonality analysis. HT was documented in 12/379 patients (3·2%) and occurred at a median time of 22 months (range; 6–202 months) after diagnosis of MALT lymphoma. By PCR‐based clonality analysis, we detected a clear‐cut clonal relationship of MALT lymphoma and diffuse large B‐cell lymphoma (DLBCL) in 8 of 11 analysed cases proving that the large majority of DLBCL following MALT lymphoma are clonally‐related and constitute a real transformation. Interestingly, HT occurred within the first 2·5 years after diagnosis in patients with clonal relationship, whereas time to aggressive lymphoma was longer in patients identified as clonally‐unrelated (most likely secondary) lymphoma (82–202 months), suggesting that HT is an early event in this disease. Survival of patients with HT was poor with 6/12 dying at 1·5–33 months after HT, however, patients with localized gastric transformation had a superior outcome with only 1/6 dying due to progression of lymphoma.

## Introduction

Representing one of the more common B‐cell malignancies, extranodal marginal zone lymphoma (MZL) of the mucosa‐associated lymphoid tissue (MALT) lymphoma accounts for approximately 8% of all newly diagnosed non‐Hodgkin lymphomas with an estimated incidence rate of 1·6/100 000 per year (Olszewski & Castillo, 2013; Cook *et al*, 2017; Jaffe *et al*, 2017). While MALT lymphoma typically presents with a benign clinical course, transformation to aggressive B‐cell lymphoma may occur and negatively impact prognosis similarly to other indolent haematological malignancies, including follicular lymphoma (FL), small lymphocytic lymphoma (SLL) or chronic lymphocytic leukaemia (CLL) (Montoto *et al*, 2007; Montoto & Fitzgibbon, 2011; Raderer *et al*, 2016). According to the literature, the rate of histological transformation (HT) to a diffuse large B‐cell lymphoma (DLBCL) has been reported to be in the range of 2–5% for MALT lymphoma, but data are inconsistent due to the fact that MALT lymphoma collectives are often published within MZL/indolent lymphoma series lacking specific subgroup analyses (Meyer *et al*, 2014; Conconi *et al*, 2015; Maeshima *et al*, 2016; Teckie *et al*, 2017). In addition, the mostly retrospective nature of the reports (partly from tertiary centres and large working parties) might have resulted in a slight selection bias with overrepresentation of HT in those collectives.

The presence of scattered blasts is usually part of the histological appearance of MALT lymphoma (Bacon *et al*, 2007; Cook *et al*, 2017; Jaffe *et al*, 2017) but the diagnosis of aggressive large cell lymphoma is established by the identification of blasts arranged in clearly identifiable diffuse sheets. However, the recent versions of the World Health Organization (WHO) classification have clearly stated that ambiguous cases should be diagnosed as DLBCL with a MALT component, rather than using the term “high grade MALT lymphoma”. This is due to the diverging clinical courses of DLBCL and MALT lymphoma, but also reflects the uncertainty of whether those cases really constitute transformation from indolent MALT lymphoma or the presence of clonally unrelated lymphomas which might have a similar underlying cause. Clinical observations have suggested that DLBCL with localized gastric presentation behave less aggressively than nodal DLBCL and might respond to eradication of *H. pylori*, suggesting a distinct entity commonly entitled “large cell variant” of gastrointestinal (GI)‐MZL (Ang *et al*, 2009; Ferreri *et al*, 2013). This concept has been underlined by comparative expression profiling of nuclear factor kappa target genes, showing a very specific expression pattern in these tissues connected to MZL rather than DLBCL (Barth *et al*, 2017).

There is, however, evidence for a clear evolutional sequence in the shift from nodal indolent to aggressive lymphoma (Montoto & Fitzgibbon, 2011). This process has been investigated for FL in particular, and is based on the assumption that pre‐programmed global changes in gene expression are a prerequisite for development of a transformed FL clone, which subsequently derives from an immature cell referred to as common progenitor or ancestor cell, but still yields, to a certain extent, intra‐clonal diversity by composite presence of transformed and non‐transformed FL cells (Carlotti *et al*, 2009; Montoto & Fitzgibbon, 2011). Currently, there is no such clear concept for MALT lymphoma but early indications for a clonal progression originate from pioneering work in the 1990ies describing “low‐grade” and “high‐grade” MALT lymphoma in gastrectomy specimens with an identical immunophenotype of both components and similar monotypic immunoglobulin expression (Chan *et al*, 1990). In addition, further data suggest *MYC* rearrangements, inactivation of *TP53* and other infrequent genetic alterations to be involved in the process of transformation (Du *et al*, 1995; Neumeister *et al*, 1997; Troppan *et al*, 2015; Raderer *et al*, 2016).

Given the lack of data on clinico‐pathological features and clonal lymphomagenesis of patients with MALT lymphoma and HT, we systematically analysed our collective of 379 patients with MALT lymphoma who were diagnosed, treated and followed at the Medical University Vienna 1999–2017 for occurrence and outcome of HT, and reassessed tissues of identified patients by polymerase chain reaction (PCR)‐based clonality analysis. As opposed to multicentric studies, our approach enabled a longitudinal assessment of patients diagnosed with MALT lymphoma over a prolonged time span, as all patients are diagnosed, staged and followed in a uniform way, thus eliminating a potential selection bias by including patients referred from other hospitals only after HT from MALT lymphoma to DLBCL.

## Methods

The objective of this analysis was to assess the frequency of HT in patients with MALT lymphoma, clinical characteristics and histological features including PCR‐based clonality analyses of both, indolent and aggressive lymphoma. Therefore, we conducted a retrospective data analysis of all patients diagnosed, treated and followed from 1999 to 2017 at the Medical University Vienna, a tertiary referral centre for patients with extranodal marginal zone B‐cell lymphoma of MALT. At the date of this analysis, a total of 379 patients with MALT lymphoma were available for inclusion in this investigation. In all patients, histological diagnosis of MALT lymphoma had been established by a reference haemato‐pathologist according to the most recent WHO classification for tumours of haematopoeitic and lymphoid tissues, including adequate immunophenotyping on paraffin‐embedded specimens i.e., CD20+CD5−CD10−cyclinD1−, demonstration of light chain restriction, assessment of plasma‐cellular differentiation and evaluation of Ki67 (Cook *et al*, 2017; Jaffe *et al*, 2017). Furthermore, fluorescence *in situ* hybridization (FISH) for detection of *t*(11;18)(q21;q21) was performed in gastric MALT lymphoma and in patients who developed aggressive lymphomas both on DLBCL as well as the MALT lymphoma. In patients with histological proof of aggressive lymphoma/DLBCL, c‐MYC, BCL2, BCL6 and MUM‐1 were also routinely analysed. The ethical board of the Medical University of Vienna had approved this investigation.

### Clonality analyses

In all patients with histologically confirmed DLBCL arising after an initial diagnosis of MALT lymphoma as assessed by re‐biopsy of progressive lesions, corresponding paraffin‐embedded tissues (both of the primary diagnosis and the time of transformation), which are routinely archived at the Department of Pathology, were further subjected to comparative clonality analysis. Clonality studies were performed following a EuroClonality validated PCR‐based multiplex protocol with the IdentiClone Gene Clonality Assays (Invivoscribe Technologies, Inc., San Diego, CA, USA), using primers targeting frameworks 1–3, the diversity and joining regions of the immunoglobulin heavy chain (*IGH*), and the variable and joining, as well as the JK‐CK intron and Kde regions of the immunoglobulin Kappa light chain (*IGK*) genes, as recently described (van Dongen *et al*, 2003; Mitterbauer‐Hohendanner *et al*, 2004). As readout systems either GeneScan (GS) fragment analysis or polyacrylamide heteroduplex (HD) analysis were used. Appropriate positive and negative controls were run simultaneously. Samples of the indolent and aggressive lymphomas of each individual patient were run in parallel, using paraffin‐embedded archived material. The clonal peaks and bands for each individual PCR product were compared and showed either similar results in all reactions, and were rated to represent identical B‐cell clones, or totally different clonal PCR products, thus representing two different lymphoma clones.

### Clinical data

Corresponding clinical data of all 379 consecutive patients were extracted from electronic and paper‐based charts. Documented data included sex, performance status, age, stage of disease at diagnosis and transformation, histological features (plasmacytic differentiation, *H. pylori* positivity on tissues), and clinical characteristics, e.g. presences of an autoimmune disease, elevated levels of lactate dehydrogenase (LDH) and beta‐2‐microglobulin (B2M). In addition, we have assessed treatment regimens (both at primary diagnosis and time of transformation). Staging was based on radiological and/or endoscopic findings according to primary site of disease (Raderer *et al*, 2016). Bone marrow biopsy was routinely performed until 2005 but afterwards restricted to cases with clinical suspicion of bone marrow infiltration due to the low detection rate in our cohort (<3%) (Raderer *et al*, 2006). Response to treatment was classified by radiological criteria or by use of Groupe d’ Etude des Lymphomes de l’ Adulte histological response criteria in the case of gastric MALT lymphoma (Copie‐Bergman *et al*, 2013). Progression‐free survival (PFS) and disease status at last follow‐up were documented. The diagnosis of transformation, i.e. presence of DLBCL/aggressive lymphoma was based on histological findings of biopsies performed in all patients with clinical suspicion of relapse following complete response (CR) after therapy and all patients with clinical suspicion of aggressive lymphoma (including rapid progression or progression at only one site with stable disease in others, occurrence of night sweat, weight loss or increase in LDH) who had only achieved partial remission (PR) or stable disease (SD) after treatment.

### Statistical analysis

Statistical analyses were performed using SPSS statistics for Windows version 25·0 (IBM, Armonk, NY, USA) and R version 3·5·2 using the packages ‘RcmdrPlugin.EZR’ (Kanda, 2013 *)* and ‚Survival’ (Therneau & Grambsch, 2000). Metric data are described using mean ± standard deviation given normal distribution or median and range in case of skewed data. For categorical data absolute frequencies and percentages are presented. Associations of binary variables were calculated using crosstabs and Fisher's exact test. Differences in means were analysed with the Student's *T*‐test. Overall survival (OS)/PFS estimations were plotted by Kaplan–Meier method. OS of transformed and non‐transformed patients was also compared using Mantel Byar test as Cox regression with time‐depending covariate. *P*‐values less than 0·05 were considered statistically significant (two‐sided).

## Results

### General population

From 1999 to 2017 a total of 379 patients with histologically verified MALT lymphoma were diagnosed and treated at the Clinical Division of Oncology, Medical University Vienna. In total, 32% of patients had gastric MALT lymphoma, while the majority of patients presented with primary extra‐gastric disease (68%) with ocular adnexal MALT lymphoma being the most common non‐gastric manifestation (24% of documented patients). The median age was 63·5 years (range; 20–90 years) and the female‐to‐male ratio was 1·4 (59% female patients, 41% male). In terms of Ann Arbor stage, 57% had localized disease without lymph node involvement (= stage IE), 21% stage IIE, 2% stage IIIE and 19% multiple site involvement i.e. stage IV. Applying the recently published MALT lymphoma prognostic index (MALT‐IPI) score (Thieblemont *et al*, 2017), 49% of patients were low risk (= no risk factor), 42% intermediate risk (= one risk factor) and 9% were high risk (= 2–3 risk factors) in patients who had complete data available (330/379). Table 1 provides more detailed characteristics of patients treated at our department.

**Table 1:** Baseline characteristics of the overall collective (*n* = 379 patients)

Gender (female/male)	222 (59%)/157 (41%)
Median age (range)	64 years (20–90 years)
Year of diagnosis	
Before 2008	196/379 (52%)
From 2008 onwards	183/379 (48%)
Primary localization of MALT lymphoma	
Gastric	123/379 (32%) (HT in 6/123, 5%)
Extra‐gastric	256/379 (68%) (HT in 6/379, 2%)
Ocular adnexa	89/379 (24%) (HT in 2/89, 2%)
Parotid gland	38/379 (10%) (HT in 2/38, 5%)
Lung	44/379 (12%) (HT in 2/44, 5%)
Other	85/379 (22%)
Bone marrow involvement	9/379 (2%)
Stage of disease	
Localized disease (Ann Arbor I‐II)	299/379 (79%)
Disseminated disease (Ann Arbor III‐IV)	80/379 (21%)
Performance status	
0	283/338 (84%)
1	42/338 (12%)
>1	13/338 (4%)
MALT‐IPI risk factors	
0	163/330 (49%)
1	137/330 (42%)
>1	30/330 (9%)
Further clinical features	
Autoimmune disorder	112/321 (35%)
Beta‐2 microglobulin > UNL	106/284 (37%)
Lactate dehydrogenase > UNL	15/334 (5%)
First line treatment	
Antibiotics	99/375 (26%)
Systemic (Immuno‐/Chemotherapy)	151/375 (40%)
Local treatment	96/375 (26%)
Wait and see	29/375 (8%)
PFS after first line treatment	53 months (95% CI 38–68 months)
Median follow‐up time (Interquartile range)	52 months (24–97 months)
Alive at last follow‐up	326/379 (86%)

HT, histological transformation; MALT, mucosa‐associated lymphoid tissue; MALT‐IPI, MALT lymphoma prognostic index; PFS, progression‐free survival; UNL, upper normal limit.

All patients with *H. pylori* positive MALT lymphoma received adequate eradication treatment (26%), the remaining patients were treated with either systemic treatment (chemo/immunotherapy, 40%) or local treatment (surgery or radiotherapy, 26%); furthermore, 8% of patients did not receive treatment and were managed by watch and wait strategy. Median estimated PFS after first line therapy was 53 months (95% confidence interval [CI] 38–68 months).

The median follow‐up time was 52 months (interquartile range 24–97 months). The estimated 5‐year and 10‐year OS for the entire cohort was 90% (95% CI 87–94%) and 81% (95% CI 75–87%) respectively. According to documented data, 4·5% of patients died due to lymphoma‐related events.

### MALT lymphoma with histological transformation

Development of aggressive B‐cell lymphoma was documented in 12 of 379 (3·2%) patients and occurred between 6·4 and 201·5 months after initial diagnosis. In addition, synchronous manifestation of MALT lymphoma and DLBCL was diagnosed in two patients with gastric MALT lymphoma and one with MALT lymphoma of the submandibular gland, constituting a total of 15 patients (4%) with documented DLBCL features at some point during the course of disease.

Of 12 patients with histologically confirmed aggressive lymphoma (eight female/four male), six initially presented with primary gastric lymphoma, two with ocular adnexal MALT lymphoma, and additional two each with pulmonary‐ and MALT lymphoma of the parotid gland, respectively, with no other extra‐gastric manifestations being registered in this subgroup. Four of these 12 patients (33%), had localized lymphoma at diagnosis, five local lymph node involvement i.e. Ann Arbor IIE (42%), one distant lymph node involvement (8%) and two patients multiple organ involvement (17%). None of these patients had evidence of *t*(11;18)(q21;q21) translocation as assessed by FISH. In terms of initial treatment for MALT lymphoma, three patients with gastric MALT lymphoma were managed with *H. pylori* eradication only, while the majority of patients had received at least one line of prior immuno‐/chemotherapy. In total, six of 12 patients (50%) experienced at least one relapse and received more than one treatment line for MALT lymphoma histology; the remaining six patients showed durable response to first line treatment for MALT lymphoma with the first/further relapse being biopsy‐proven DLBCL. There was no difference in relapse rates for MALT lymphoma in patients with subsequent aggressive histology if compared to the general collective (50% vs. 45%, *P* = 0·776). See Table 2 for detailed clinical data.

**Table 2:** Clinical Characteristics of patients with MALT lymphoma and histological transformation (*n* = 12)

ID	Sex	Age (years)	Primary localization	Stage	MALT‐IPI	Treatment for MALT lymphoma before transformation (best response)	Time to transformation (months)	Localization at transformation	Treatment for transformed lymphoma (best response)	Current status	PFS^a^	OS (OS2^b^)
1	F	52	Stomach	I	0	HP‐eradication (PD), R‐2CDA (CR), lenalidomide (transformation)	25	Stomach	R‐CHOP (CR)	Ongoing remission	84+	109+ (85+)
2	F	64	Stomach	II	1	R‐lenalidomide (CR)	22	LNN (bulky disease), *supra*‐ and infradiaphragmatic	DA‐R‐EPOCH (PD), bortezomib (PD)	Died due to progression of lymphoma	9	33 (12)
3	F	72	Ocular adnexa	II	1	Radiotherapy (PD), everolimus (PD)	15	LNN, spleen, bone marrow	Patient deceased	Died due to progression of lymphoma	–	15 (0)
4	F	58	Ocular adnexa, gluteal, LNN	IV	1	Doxycycline (PD), radiotherapy (PR), BR (PD)	19	LNN cervical	R‐COMP (PD), radiotherapy (PD)	Died due to progression of lymphoma	2	22 (3)
5	M	66	Stomach	II	0	HP‐eradication (PR)	6	Stomach, LNN *supra*‐ and infradiaphragmatic	R‐CHOP (CR)	Alive with MALT lymphoma	15 (MALT)	53+ (46+)
6	M	56	Stomach	III	0	BR (CR), HP‐eradication (SD)	31	Stomach, LNN	R‐COMP (CR)	Ongoing remission	13+	44+ (13+)
7	M	68	Stomach	II	0	HP‐eradication (SD)	17	Stomach, LNN *supra*‐ and infradiaphragmatic	R‐CHOP + 2 × R mono (CR transformed but residual gastric MALT lymphoma)	Alive with MALT lymphoma	13+	31+ (14+)
8	F	70	Stomach	I	1	HP‐eradication (PR)	10	Stomach	R‐CHOP (PR, residual gastric MALT lymphoma)	Alive with MALT lymphoma	33+	44+ (34+)
9	M	53	Lung (bilateral)	IV	1	Thalidomide (SD), R‐2CDA (CR)	202	Stomach	DA‐R‐EPOCH (CR)	Ongoing remission	16+	218+ (16+)
10	F	55	Lung	II	1	CHOP (CR), rituximab (PR), ofatumumab (CR)	194	Lung bilateral, LNN disseminated	DHAP + MTX intrathecal (patient deceased)	Died due to progression of lymphoma	1	196 (2)
11	F	51	Parotid gland	I	0	COP (CR)	82	Stomach, LNN	R‐CHOP (PR), Radiation of LNN	Died due to progression of lymphoma	15	97 (16)
12	F	69	Parotid gland	I	0	R‐FCM (CR)	22	Parotid gland, bone marrow	R‐CHOP (CR), Radiation	Died due to progression of lymphoma/MDS	17	55 (33)

2CDA, cladribine; BR, bendamustine, rituximab; CHOP, cyclophosphamide, doxorubicin, vincristine and prednisone; COMP, cyclophosphamide, vincristine, non‐pegylated liposomal doxorubicin, prednisone; COP, cyclophosphamide, vincristine, prednisone; CR, complete remission; DA, dose‐adjusted; DHAP, dexamethasone, high dose cytarabine, cisplatin; EPOCH, etoposide, prednisone, vincristine, cyclophosphamide, doxorubicin; F, female; FCM, fludarabine, cyclophosphamide, mitoxantrone; HP, *Helicobacter pylori*; ID, identification number; LNN, lymph nodes; M, male; MALT, mucosa‐associated lymphoid tissue; MALT‐IPI, MALT lymphoma prognostic index; MDS, myelodysplastic syndrome; OS, overall survival; PD, progressive disease; PFS, progression‐free survival; PR, partial remission; R, rituximab; SD, stable disease.

a Progression free survival after treatment start for transformed lymphoma.

b Overall survival after histological transformation

Median time to HT of lymphoma was 22 months (range; 6·4–201·5) in these 12 patients. At the time of diagnosis of aggressive lymphoma, three patients with initial gastric MALT lymphoma had localized disease, whereas the remaining patients presented with advanced disease (IIE *n* = 3, IIIE *n* = 3, IV *n* = 3). While the site of DLBCL was identical to the initial localization in 7 patients (gastric *n* = 5, lung *n* = 1, parotid gland *n* = 1), three patients showed lymph node involvement in particular (one with gastric primary, two with orbital primary), and one primary MALT lymphoma of the lung and parotid gland each, developed DLBCL in the stomach. With the caveat of the small number of patients with HT, there was a non‐significant trend towards higher MALT‐IPI scores (i.e. intermediate/high risk) in patients developing HT (*P* = 0·135), however, there was no difference in terms of primary localization, i.e. gastric versus extra‐gastric (*P* = 0·215), elevation of B2M (*P* = 0·301), plasmacytic differentiation (*P* = 0·728), presence of an autoimmune disease (*P* = 0·340), mean age at diagnosis (*P* = 0·951) and the year of diagnosis, based on a cut‐off in the middle of the recruitment period i.e. before 2008 versus from 2008 onwards (*P* = 0·564).

Remarkably, at the time of DLBCL‐diagnosis, 8 of 12 patients (67%) had radiological signs of local or distant lymph node involvement (irrespective of the presence of multiple extranodal sites), and a total of 6 of 12 (50%) patients already had lymph node involvement at primary diagnosis. This is opposed to a low rate of lymph node involvement present in only 11% (42/367) of the general collective (both *P*‐values <0·001), suggesting nodal infiltration with MALT lymphoma as a significant risk factor in this context. Also, though being a rare event in general, bone marrow infiltration at any point of disease was significantly more common in patients developing DLBCL (17% vs. 2%, *P* = 0·029).

### Treatment and prognosis

**Figure 1:**
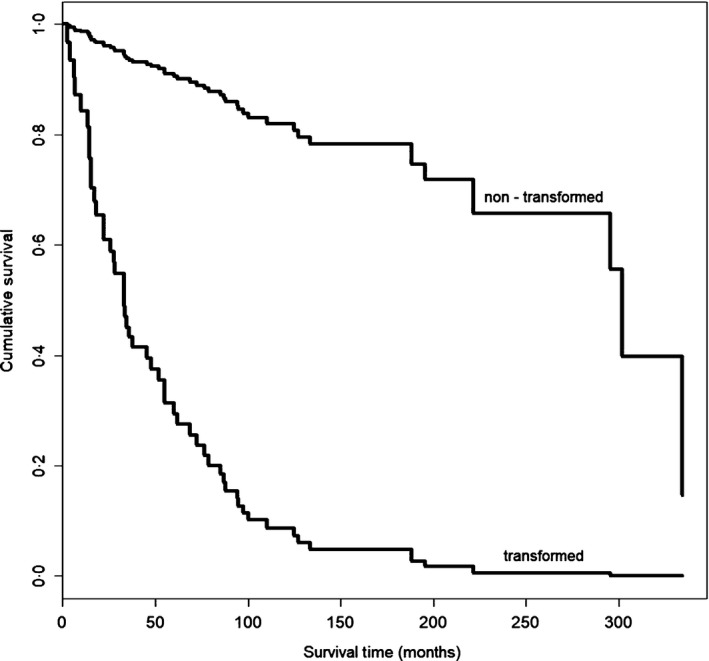
Cox regression survival curve for estimated overall survival in histological transformed (corrected for delayed entry) and non‐transformed MALT lymphoma patients, respectively. *X*‐axis: follow‐up in months, *y*‐axis: cumulative survival.

Treatment for DLBCL was anthracycline‐based in 10/12 (R‐CHOP [rituximab, cyclophosphamide, doxorubicin, vincristine and prednisone]/R‐COMP [rituximab, cyclophosphamide, vincristine, non‐pegylated liposomal doxorubicin, prednisone] *n* = 8; dose adjusted R‐EPOCH [rituximab, etoposide, prednisone, vincristine, cyclophosphamide, doxorubicin] *n* = 2), one patient with loss of CD20 expression was treated with DHAP (dexamethasone, high dose cytarabine, cisplatin), and one died due to rapid lymphoma progression before initiation of specific treatment. Eight of eleven patients responded to chemotherapy, while two patients were refractory to the first line and salvage approach (radiotherapy = 1, bortezomib = 1) and one died during initial treatment (no response assessment available). Median PFS after treatment start for DLBCL was 15·0 months (95% CI 11–18). In general, survival was poor, with 6/12 (50%) dying due to progression of lymphoma at 1·5–33 months after documentation of DLBCL. Comparison of survival curves using Mantel Byar test confirmed that OS of patients developing DLBCL was significantly worse independent of time to transformation (Hazard Ratio = 12·282, 95% CI [4·99–29·54]; χ²_(df=1) _= 51·452; *P* < 0.001). See Fig. 1 for corresponding survival curves.

Interestingly, patients with DLBCL following primary gastric MALT lymphoma appeared to have a better outcome than patients with DLBCL documented at an extra‐gastric site, with only one of six patients with gastric lymphoma dying from DLBCL, but five of six with extra‐gastric primary. Equally, six of seven patients with stomach involvement at the time of transformation survived, while all patients with non‐GI lymphoma died of lymphoma‐related causes.

Noteworthy, in three patients with transformed gastric MALT lymphoma, a MALT lymphoma remnant/relapse of MALT lymphoma component without evidence for DLBCL has been documented in the further course of disease. Currently, all of these patients are without signs of progression and managed with a watch and wait strategy.

### Clonality analysis and histological features

**Figure 2:**
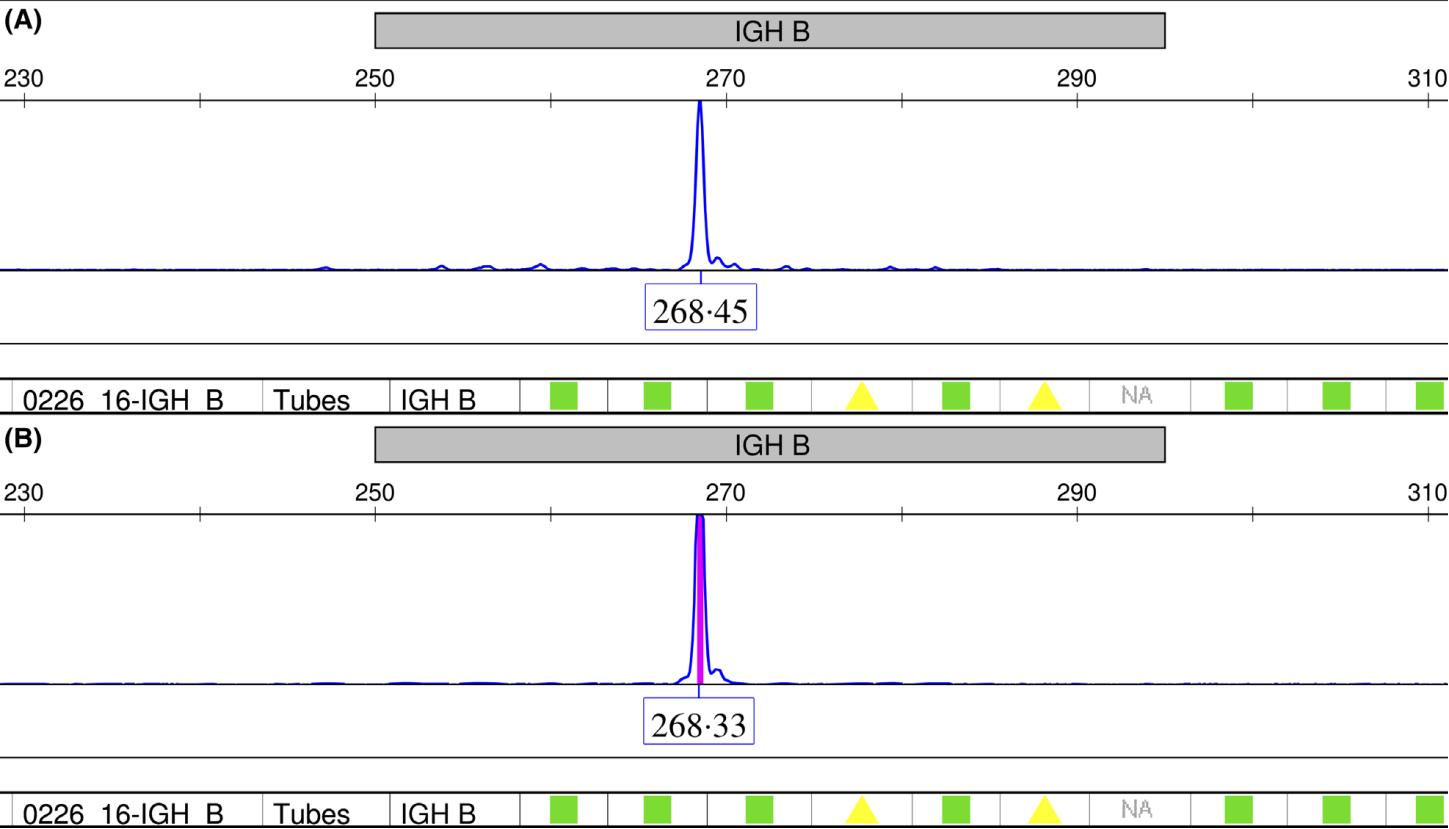
Polymerase chain reaction‐based clonality analysis showing identical peaks indicating clonal rearrangements of the FR2 region of *IGH* in (A) primary indolent MALT lymphoma and (B) in the histologically confirmed transformation to aggressive diffuse large B‐cell lymphoma (Patient 2). [Colour figure can be viewed at wileyonlinelibrary.com]

The MALT and DLBCL components of a total of 11 patients could be analysed in parallel, whereas no tissues were available in one patient. For site of biopsies and more detailed results see Table 3. Seven patients showed identical clonal peaks or bands in both MALT as well as in their aggressive lymphoma, suggesting a clonal relationship and thus transformation from MALT lymphoma (see Fig. 2 depicting an example of identical clonal peaks in both MALT and aggressive lymphoma). One additional patient showed both monoclonal *IGH* and *IGK* rearrangement in the MALT lymphoma, whereas the monoclonal *IGH* rearrangement could not be detected in the aggressive tumour developing 1 year after diagnosis of MALT lymphoma. However, the clonal relationship could be demonstrated by detection of an identical *IGK* gene rearrangement. Two further patients showed clearly different clones for both *IGH* and *IGK* in the MALT lymphoma and in the subsequent DLBCL, pointing to clonally unrelated lymphoma entities. In the last patient, no clonal rearrangement for both *IGH* and *IGK* could be detected in the initial MALT lymphoma‐specimen, whereas a clonal rearrangement for both *IGH* and *IGK* could be demonstrated in the subsequent DLBCL. A definitive proof of clonal relationship between these two tumours therefore could not be demonstrated. Thus, a clear clonal relationship between primary MALT lymphoma and DLBCL was found in 8 of 11 analysed patients, suggesting clear‐cut HT from MALT lymphoma in those cases, while in two patients the aggressive lymphoma was found to be a clonally unrelated, secondary lymphoma and one case could not be clearly classified. Notably, HT occurred within the first 2·5 years after diagnosis in all patients with a clonal relationship, whereas time to occurrence of aggressive lymphoma was notably longer in patients identified as secondary (clonally‐unrelated) lymphoma (range; 82–202 months), suggesting that HT is an event occurring relatively early in the course of disease.

**Table 3:** Results of clonality analysis of patients with MALT lymphoma and aggressive lymphoma

	Biopsy site 1 (initial diagnosis)	Biopsy site 2 (transformation)	Histological diagnosis	*IGH*	*IGK*	Clonality analysis
1	Stomach	Stomach	MALT lymphoma Diffuse large B‐cell lymphoma	+, id +, id	− −	Identical clone
2	Lymph node (abdominal)	Lymph node (abdominal)	MALT lymphoma Diffuse large B‐cell lymphoma (double expressor – *MYC*,* BCL2*)	+, id +, id	+, id +, id	Identical clone
3	Ocular adnexa	Lymph node (cervical)	MALT lymphoma Diffuse large B‐cell lymphoma	+, id +, id	− −	Identical clone
4	Ocular adnexa	Lymph node (cervical)	MALT lymphoma, CD5‐ Diffuse large B‐cell lymphoma, CD5+	+, id +, id	+, id +, id	Identical clone
5	Stomach	Stomach	MALT lymphoma Diffuse large B‐cell lymphoma	+, id +, id	+, id +, id	Identical clone
6	Lymph node (cervical)	Stomach	MALT lymphoma Diffuse large B‐cell lymphoma	+, id +, id	+, id +, id	Identical clone
7	Stomach	Lymph node (cervical)	MALT lymphoma Diffuse large B‐cell lymphoma	+, id +, id	+, id +, id	Identical clone
8	Stomach	Stomach	MALT lymphoma Diffuse large B‐cell lymphoma	+ −	+, id +, id	Identical clone
9	Lung	Stomach	MALT lymphoma Diffuse large B‐cell lymphoma, *MYC*+ (FISH)	− +	− +	Secondary lymphoma
10	Lung	Lung	MALT lymphoma Diffuse large B‐cell lymphoma, CD20‐, *MYD88* mutation (L265P)	+, id +, id	+, id +, id	Secondary lymphoma
11	Parotid gland	Stomach	MALT Lymphoma Diffuse large B‐cell lymphoma, Burkitt‐like	+, id +, id	+, id +, id	Secondary lymphoma
12	Parotid gland	Parotid gland	MALT Lymphoma Diffuse large B‐cell lymphoma	No data No data	No data No data	No data No data

d, different; FISH, fluorescence *in situ* hybridization; id, identical; *IGH*, immunoglobulin heavy chain; *IGK*, immunoglobulin Kappa light chain; MALT, mucosa‐associated lymphoid tissue.

In terms of further histological/molecular features of DLBCL, one patient with clonally related lymphoma was a double expressor of *MYC* and *BCL2*, and one presented with CD5+ aggressive lymphoma following an initially CD5− MALT lymphoma. The two patients with clonally unrelated lymphoma both presented with high risk features (one with a *MYD88* mutation and one with a Burkitt‐like phenotype of DLBCL). The remaining patients did not show any specific alterations and no patient with gastric involvement was *H. pylori* positive at the time of transformation.

## Discussion

The aim of the present study was to evaluate the incidence of true HT, pathological features, clinical characteristics and prognosis of patients with DLBCL following extranodal B‐cell lymphoma of MALT lymphoma in a large collective of patients treated at the Medical University Vienna. With 379 subjects included, this is – to the best of our knowledge – the most extensive single‐centre investigation with standardized follow‐up addressing this issue in a collective consisting exclusively of MALT lymphoma patients. Whereas general features may be extrapolated from mixed MZL populations also including splenic and nodal MZL, it has repeatedly been shown that MALT lymphoma presents with a distinct biological and also molecular profile requiring an individual work‐up of this specific entity (Bertoni *et al*, 2018). While our data have to be interpreted within the caveats of retrospective analyses, we provide both extensive clinical data with an unbiased longitudinal follow‐up of patients along with clonality analysis by PCR‐based testing on both primary indolent and subsequent aggressive lymphoma, thus offering a unique insight into pathogenesis of this event.

The natural course of MALT lymphoma is favourable and, despite more than 40% progressing after initial therapy, treatment at first and further relapse is also associated with acceptable response rates and good overall outcome (Kiesewetter *et al*, 2015; Raderer *et al*, 2016). However, and in line with other indolent lymphoma entities, transformation to aggressive lymphoma may occur and drastically worsen the prognosis. According to the literature, the rate of transformation in MALT lymphoma is 2–5% but most data derive from limited retrospective series or mixed MZL collectives (Meyer *et al*, 2014; Conconi *et al*, 2015; Maeshima *et al*, 2016; Teckie *et al*, 2017). To date, the largest retrospective analysis addressing HT and outcome in mixed MZL was reported by Conconi *et al* (2015) and included 157 patients with MALT lymphoma. The HT rate in this series was 4% (6/157) and 4/6 (66%) patients died between 1 and 96 months post‐HT. Furthermore, an Asian series of 467 MALT lymphoma patients reported a DLBCL rate of 8% but more than two‐thirds of patients had synchronous manifestations of MALT and DLBCL. The rate of DLBCL in the course of the disease was, however, in a range comparable to our series and was 3%, 3% and 5% at 5, 10 and 15 years, respectively (Maeshima *et al*, 2016). In contrast to our data, none of these series attempted to characterise the clonal relationship, though the term HT was used in all series.

In addition to retrospective data, two long‐term updates of major first line studies have recently been published, presenting also documentation of metachronous DLBCL along with PFS (Salar *et al*, 2017; Zucca *et al*, 2017;). Both studies exclusively included patients with MALT lymphoma, obviating bias by mixed MZL histologies. In the International Extranodal Lymphoma Study Group (IELSG)‐19 trial (chlorambucil ± rituximab); a median follow‐up of 7·4 years was presented and DLBCL was reported in 10/401 patients (2·5%), with six patients dying during the observation period (Zucca *et al*, 2017). In the Spanish phase II trial MALT2008‐01, rituximab‐bendamustine was assessed as upfront strategy in a total of 60 patients (Salar *et al*, 2017). In this series, the DLBCL rate was 5% at 7+ years with no data on DLBCL‐related deaths being presented. While the influence of respective treatment/trial inclusion criteria can only be speculated on and the absolute numbers of patients with DLBCL are small, the data again suggest a poor outcome for this subgroup.

In the current analysis, we present 379 patients with MALT lymphoma, including 12 patients developing DLBCL (3·2%). While at diagnosis, patients with aggressive lymphoma in the course of disease did not differ from patients not experiencing DLBCL in terms of age, primary localization, plasmacytic differentiation and autoimmune disease (*P*‐values all non‐significant), it was striking that patients experiencing HT had a significantly higher rate of lymph node involvement (67% vs. 11%, *P* < 0·001), clearly suggesting this as a relevant “red flag” in MALT lymphoma. Interestingly, lymphadenopathy has already been proposed as a negative predictive marker for OS, PFS and cause‐specific survival in a series of 180 patients with non‐gastric MALT lymphoma but had not yet been associated with HT (Zucca *et al*, 2003). In addition, bone marrow infiltration at any point was significantly more common in patients with HT (17% vs. 2%, *P *=* *0·029). However, due to the fact that the rate of bone marrow infiltration is below 3% in our collective (see Table 1) and that there are currently no therapeutic consequences as has been shown for gastric MALT lymphoma in particular (Choi *et al*, 2018), we do not recommend bone marrow biopsy on a routine basis.

One focus of our analysis was PCR‐based clonality analysis in patients developing DLBCL to assess the rate of “true” HT as opposed to the development of a clonally unrelated secondary lymphoma due to potential similar risk factors, such as *H. pylori*‐infection. Based on recent high‐resolution genomic profiling of 28 GI‐MALT lymphomas (small cell/small cell but with large cell areas/large cell morphology), it appears that in MZL genomic complexity evolves over time, and gains of specific proto‐oncogenes including *REL*,* BCL11A*,* ETS1*,* PTPN1*,* PTEN* and *KRAS* are exclusively found in large cell variants, suggesting these to be involved in a distinct transformation process, while other aberrations are found in all three groups, underlining a clonally‐related origin of lymphoma (Barth *et al*, 2001; Flossbach *et al*, 2013). Furthermore, it has been shown that the copy numbers/status of several genes does change over time and that they are particularly diverse in MALT lymphomas also showing areas of large cells, potentially indicating a selection of sub‐clones over time.

To determine if two lymphomas evolving at different timepoints are clonally related, a PCR‐based fragment analysis of *IGH* and *IGK* gene rearrangements is usually performed. By means of this approach, we could clearly demonstrate that there was a straight clonal relationship of indolent MALT lymphoma and aggressive lymphoma in 8 of 11 patients, thus confirming HT and an evolutional, clonal process from MALT lymphoma. Interestingly, all patients presenting with an identical clone developed HT within 30 months after diagnosis of MALT lymphoma. In contrast, lymphomas showing different rearrangements occurred noticeably later and up to 200 months after diagnosis. These findings suggest that classical HT might constitute a relatively early event in the course of the disease as opposed to former case series, suggesting a time span of more than a decade between these events (Yoshino *et al*, 2000), which may more likely represent a secondary independent DLBCL development. A potential pitfall in our PCR‐based approach may be the limitation of the method with regard to potential somatic hypermutation because only large insertions or deletions, but no subtle differences such as single point mutations, will be detected by this method. Intraclonal sequence variation characterizing ongoing mutation is a feature of normal germinal centre derived B‐cells and is also a typical feature of several B‐cell lymphomas, especially FL and some DLBCL (Klein *et al*, 1998; Lossos *et al*, 2000). Ongoing mutation (although to a comparatively low level) has also been shown to be a feature of MALT lymphoma driven by antigen‐stimulation (Du *et al*, 1996). Conceivably, it cannot be ruled out that the lymphomas showing different clonal rearrangements in our series may still be ontogenetically related, and are composed of different clones with a common origin, as has been demonstrated by sequence analysis in a series of various relapsed lymphomas (Geurts‐Giele *et al*, 2013). Furthermore, it cannot completely be ruled out that a relapsed, apparently differently rearranged lymphoma may be the result of a dominant proliferation of a subclone of the primary lymphoma, as has been described in FL (Aarts *et al*, 2001).

In terms of clinical outcome, overall prognosis after HT and development of DLBCL, respectively, was poor with six of 12 patients dying lymphoma‐related at 1·5–33 months following documentation of aggressive lymphoma. All patients were scheduled to receive salvage chemotherapy; but one patient did not start treatment due to rapid progression and subsequent death. Consequently, we have to conclude that despite the fact that anthracycline‐based chemotherapy is the standard of care in this situation, it does not appear to be sufficiently effective. However, it must be highlighted that patients with gastric HT, i.e. clonally‐related DLBCL, and no signs of further extranodal involvement had a better prognosis, again underlining the concept of “large‐cell” GI‐MZL. Also, the idea of only selected sub‐clones developing HT is encouraged by three patients with gastric MALT lymphoma who achieved complete response of the high‐grade component following anthracycline‐based treatment but presenting with a residual and clinically typical MALT lymphoma remnant that had not been detected before. The additional use of DNA‐sequencing methods might provide more information in terms of defining genetic events and evolution of sub‐clones, but is difficult due to the lack of sufficient tissues in most cases of MALT lymphoma.

With the caveats of the retrospective nature of this analysis, we present data from the so far largest collective of MALT lymphoma patients evaluated in a single centre setting. The clonality analyses performed in primary MALT and aggressive lymphomas underline the direct clonal relationship in most cases and consequently support the notion of direct transformation in a small percentage of patients with MALT lymphoma.

## Author contributions

Conception and design of the study: BK, MR, ISK; Performed research: BK, WL, AIS, CK, GB, ISK, MR; Patient management/data acquisition: BK, WL, WD, JL, MEM, MR; Performed data analysis: BK, WL, WD, JL, MEM, MW, AIS, CK, GB, ISK, MR; Wrote manuscript: BK, ISK, MR; Approved final manuscript: BK, WL, WD, JL, MEM, MW, AIS, CK, GB, ISK, MR.

## Disclosures

The authors have declared no conflicts of interest.
